# Edaravone Protects against Methylglyoxal-Induced Barrier Damage in Human Brain Endothelial Cells

**DOI:** 10.1371/journal.pone.0100152

**Published:** 2014-07-17

**Authors:** Andrea E. Tóth, Fruzsina R. Walter, Alexandra Bocsik, Petra Sántha, Szilvia Veszelka, Lajos Nagy, László G. Puskás, Pierre-Olivier Couraud, Fuyuko Takata, Shinya Dohgu, Yasufumi Kataoka, Mária A. Deli

**Affiliations:** 1 Institute of Biophysics, Biological Research Centre of the Hungarian Academy of Sciences, Szeged, Hungary; 2 Avidin Ltd., Szeged, Hungary; 3 Inserm, U1016, Institut Cochin, Paris, France; 4 CNRS, UMR8104, Paris, France; 5 Université Paris Descartes, Sorbonne Paris Cité, Paris, France; 6 Department of Pharmaceutical Care and Health Sciences, Fukuoka University, Fukuoka, Japan; University of Sassari, Italy

## Abstract

**Background:**

Elevated level of reactive carbonyl species, such as methylglyoxal, triggers carbonyl stress and activates a series of inflammatory responses leading to accelerated vascular damage. Edaravone is the active substance of a Japanese medicine, which aids neurological recovery following acute brain ischemia and subsequent cerebral infarction. Our aim was to test whether edaravone can exert a protective effect on the barrier properties of human brain endothelial cells (hCMEC/D3 cell line) treated with methylglyoxal.

**Methodology:**

Cell viability was monitored in real-time by impedance-based cell electronic sensing. The barrier function of the monolayer was characterized by measurement of resistance and flux of permeability markers, and visualized by immunohistochemistry for claudin-5 and β-catenin. Cell morphology was also examined by holographic phase imaging.

**Principal Findings:**

Methylglyoxal exerted a time- and dose-dependent toxicity on cultured human brain endothelial cells: a concentration of 600 µM resulted in about 50% toxicity, significantly reduced the integrity and increased the permeability of the barrier. The cell morphology also changed dramatically: the area of cells decreased, their optical height significantly increased. Edaravone (3 mM) provided a complete protection against the toxic effect of methylglyoxal. Co-administration of edaravone restored cell viability, barrier integrity and functions of brain endothelial cells. Similar protection was obtained by the well-known antiglycating molecule, aminoguanidine, our reference compound.

**Conclusion:**

These results indicate for the first time that edaravone is protective in carbonyl stress induced barrier damage. Our data may contribute to the development of compounds to treat brain endothelial dysfunction in carbonyl stress related diseases.

## Introduction

Increased serum levels of reactive carbonyl species, such as methylglyoxal, are present in several pathologies and cause complications in severe conditions and diseases, like diabetes mellitus [1], [2], cardiovascular diseases [3], [4], atherosclerosis [5], hypertension [6], metabolic syndrome [7], obesity [8], psoriasis [9], aging [10], [11] Alzheimer’s disease [12] [13], dementias [14], and other neurobiological diseases [15]. Methylglyoxal is a highly reactive α-oxoaldehyde with strong oxidant and glycation properties [16]. Its immediate elimination by detoxification systems is crucial [17]. Accumulated methylglyoxal reacts with proteins, DNA and other biomolecules [18] causing inhibition of enzyme activity [19], transcriptional activation [20], apoptosis [21]. The end products of the reactions between methylgyoxal and free amino groups of molecules are insoluble protease-resistant polymers (advanced glycation end products AGE) [22]. Methylglyoxal triggers carbonyl [18] and oxidative stress [23], [24] and activates a series of inflammatory responses leading to accelerated vascular endothelial damage [25]–[27].

Based on data obtained on peripheral endothelial cells, the effect of methylglyoxal on brain microvascular endothelium, which forms the blood-brain barrier was also investigated [25], [28]. A concentration-dependent cell toxicity and barrier dysfunction was recently described on a brain endothelial cell line [28]. This study reported methylglyoxal-induced glycation of the tight junction protein occludin in culture, as well as in brain microvessels of diabetic rats, and a disturbed architectural organization of zonula occludens-1 protein. Similar to other cellular systems, methylglyoxal-treatment promoted carbonyl and oxidative stress in brain endothelial cells [28]. Methylglyoxal induced mitochondrial apoptotic signaling: decreased mitochondrial membrane potential, activated caspases and perturbed the cellular glutathione redox status [25]. These findings indicate that methylglyoxal-induced carbonyl and oxidative stress may play an important role in neurovascular pathology, and brain endothelium can be an early and significant target site of methylglyoxal.

The prevention of methylglyoxal-induced injury is in the focus of current research [29]. Aminoguanidine was the first drug extensively studied, and attenuated the development of a range of diabetic vascular complications both in vitro and in vivo. However, due to toxic side effects at high doses, it failed in clinical trials. This compound is considered as a prototype for antiglycation agents and used as a reference molecule in experiments [30]. Recently, a new promising agent, edaravone is investigated for its beneficial effects on brain endothelial cells. Edaravone is a neuroprotective free radical scavenger. It is the active substance of a Japanese medicine, which helps neurological recovery following acute brain and subsequent cerebral infarct [31], [32]. To further reveal the mechanism of protection, brain microvessels [33] and the blood-brain barrier [34] were investigated as potential pharmaceutical targets of edaravone in animal models of stroke. The effect of edaravone alone has been described on barrier function: it promoted tight junction formation via activation of sphingosin-1-phospate signaling pathway [35] and down-regulation of interleukin-1β induced monocyte chemoattractant protein-1 secretion [36] in human microvascular endothelial cells. In a recent study, methylglyoxal-induced decrease in cell viability and methylglyoxal enhanced cell injury by oxygen-glucose deprivation were alleviated by pretreatment with edaravone in brain endothelial cells [37]. However, it remained unanswered whether edaravone can also protect against methylglyoxal-induced barrier dysfunction of brain endothelial monolayers.

The tight intercellular barrier maintaining low permeability is the fundamental characteristic of brain endothelial cells [38]. Therefore, this study aimed to clarify the effect of edaravone against methylglyoxal-induced barrier and morphological damage. In the experiments the widely used human hCMEC/D3 brain endothelial cell line [39], and new investigation methods, such as impedance monitoring in multiwell plates and holographic phase contrast imaging were used in addition to viability assays, permeability tests and immunohistochemistry for junctional proteins.

## Materials and Methods

### Ethics statement

All procedures involving experimental animals adhered to the law (No. 105) and notification (No. 6) of the Japanese Government, and were approved by the Laboratory Animal Care and Use Committee of Fukuoka University. The details of the isolation of primary brain endothelial cells from rats is described in Text S1.

### Materials

All reagents were purchased from Sigma-Aldrich Ltd., Hungary, unless otherwise indicated.

### Cell Culture

Human hCMEC/D3 brain endothelial cell line [39], [40] at passage number ≤35 was used in the experiments. Cells were plated on rat tail collagen-coated culture dishes (Orange Scientific, Braine-l’Alleud, Belgium) or Transwell clear inserts (polyethylene membrane, 0.4 µm pore size, 1.2 cm^2^ surface area, Corning Life Sciences, Tewksbury, MA, USA) depending on the experiment and cultured at 37°C, 5% CO_2_ in EBM-2 medium (Lonza, Basel, Switzerland) containing 5% fetal bovine serum, hydrocortisone (1.4 µM), 10 mM HEPES, gentamycin (50 µg/mL), acid ascorbic (5 µg/mL), 1% chemically defined lipid concentrate, basic fibroblast growth factor (1 ng/mL) (Roche, Basel, Switzerland). Cells were seeded in culture dishes at a density of 2.5×10^4^ cells/cm^2^ and the medium was changed every 3 days. At the first change of medium, the medium was supplemented with 10 mM lithium chloride [41]. When cells reached approximately 80–90% of confluence in the dish, they were subcultured with 0.05% trypsin-EDTA solution. For the cytotoxicity assays cells were cultured in 96-well culture plates (Orange Scientific, Braine-l’Alleud, Belgium). For real-time cell electronic sensing 96-well plates with gold electrodes (E-plate 96, ACEA Biosciences, San Diego, USA) were used. For permeability studies cells were cultured on Transwell inserts.

### Treatments

Human brain endothelial cells were treated with methylglyoxal in the 100–1000 µM concentration range in EBM-2 medium containing 10% fetal bovine serum, HEPES (10 mM) and gentamycin (50 µg/mL). Edaravone was used in the 600–3000 µM concentration range. Aminoguanidine, a well-known antiglycation agent, was tested at 600–2000 µM concentration and applied as a positive control [42]. Triton X-100 detergent was used at 10 mg/ml concentration in viability assays as a reference compound to cause cell death.

### Real-Time Monitoring of Impedance

Impedance-based cell electronic sensing is a label-free technique for dynamic monitoring of living cells. The RTCA-SP instrument (ACEA Biosciences, Inc., USA) utilizes an automatical and continuous impedance measurement to non-invasively quantify adherent cell proliferation and viability in real-time. This method has been succesfully used to measure number, adherence, growth and health of cells in control and treatment conditions [43]–[46]. E-plates were coated with rat tail collagen at room temperature and dried for 40 min under UV and air-flow. Culture medium (50 µL) was added to each well for background readings, then 50 µL of cell suspension was dispensed at the density of 1.6×10^4^ cells/well. The cells were kept in incubator at 37°C for 5 days until reaching confluence. Impedance was monitored every 2 minutes. The cell index at each time point was defined as (Rn - Rb)/15, where Rn is the cell-electrode impedance of the well when it contains cells and Rb is the background impedance of the well with the medium alone.

### MTT Dye Reduction Cell Viability Assay

Living cells convert the yellow dye 3-(4,5-dimethyltiazol-2-yl)-2,5-diphenyltetrazolium bromide (MTT) to purple, insoluble formazan crystals. Decrease in dye conversation reflects cellular damage. Human endothelial hCEMC/D3 cells grown to confluency in 96-well culture plates were treated with methylglyoxal with or without protective agents for 8 hours. Then the treatment medium was removed from the wells and the cells were incubated with MTT solution (0.5 mg/mL) in Dulbecco’s modified Eagle’s medium for 3 hours in CO_2_ incubator. The amount of formazan crystals was dissolved in dimethyl-sulfoxide and determined by measuring absorbance at 570 nm wavelength with a microplate reader (Fluostar Optima, BMG Labtechnologies, Germany). Cells receiving treatment medium without methylglyoxal or protective agents, the control group, was considered as 100% viable.

### Detection of Reactive Oxygen Species

To measure reactive oxygen species (ROS) we used a fluorometric detection probe, chloromethyl-dichloro-dihydro-fluorescein diacetate (DCFDA) (Molecular Probes, Life Technologies Corp., Carlsbad, USA). This indicator penetrates the cells by diffusion and becomes deacetylated by intracellular esterases. Oxidation of DCFDA by reactive oxygen species yields a fluorescent molecule. Confluent brain endothelial cell layers cultured in 96-well plates were pretreated with methylglyoxal (600 µM) and/or protective agents for 0–8 hours, then washed, and incubated with Ringer–Hepes buffer containing 2 µM DCFDA and 1.5 µM pluronic acid (Life Technologies, Molecular Probes, USA) for 1 hour at 37°C. Hydrogen peroxide pretreatment (100 µM, 30 min) served as a positive control in the ROS assay. The plates were measured by Fluostar Optima fluorescent plate reader (BMG Labtechnologies, Germany) at 485 nm excitation and 520 nm emission wavelengths. The fluorescent values were presented as percent of the control group (cells receiving treatment medium without methylglyoxal or protective agents) after 1 hour incubation with DCFDA indicator.

### Measurement of Barrier Functions: Transendothelial Electrical Resistance

Transendothelial electrical resistance (TEER) reflects the permeability of intercellular tight junctions for ions. TEER was measured by an EVOM resistance meter using STX-2 electrodes (World Precision Instruments Inc., Sarasota, FL, USA) and expressed relative to the surface area (Ω×cm^2^). TEER values of cell-free inserts were subtracted from the measured data. The TEER of untreated human hCMEC/D3 brain endothelial cell monolayers was 43.3±6.1 Ω×cm^2^ (mean ± SD; n = 60) in agreement with literature data [40]. Resistance measurements were carried out before and after treatments to check the barrier integrity. The TEER values are presented as percent of non treated control groups.

### Measurement of Barrier Functions: Permeability Experiments

To measure the flux of permeability marker molecules fluorescein isothiocyanate labeled dextran (FITC-dextran, mw: 4.4 kDa) and Evans blue-labeled albumin (mw: 67 kDa) across endothelial cell layers hCMEC/D3 cells were seeded onto Transwell inserts and grown for 5 days. Inserts with confluent layers were transferred to 12-well plates containing 1.5 mL Ringer–Hepes solution (118 mM NaCl, 4.8 mM KCl, 2.5 mM CaCl_2_, 1.2 mM MgSO_4_, 5.5 mM d-glucose, 20 mM Hepes, pH 7.4), which was the abluminal (lower) compartment in the permeability experiments. In the upper chambers (luminal compartment) culture medium was replaced by 500 µL of Ringer-Hepes buffer containing 100 µg/mL FITC-dextran solution and 165 µg/mL Evans blue bound to 0.1% BSA. The plates were kept in a 37°C incubator with 5% CO_2_ on a horizontal shaker (100 rpm) for 1 h. After incubation the concentrations of the marker molecules in samples from the luminal (upper) and abluminal (lower) compartments were determined by a fluorescent microplate reader (Fluostar Optima; emission wavelength: 485 nm, excitation wavelength: 520 nm). Flux across cell-free inserts was also measured.

The apparent permeability coefficient (P_app_) was calculated from the concentration difference of the tracer in the abluminal compartments (Δ[C]_A_) after 1 hour and luminal compartments at 0 hour ([C]_L_), the volume of the abluminal compartment (V_A_; 1.5 mL) and the surface area available for permeability (A; 1.1 cm^2^) by the following equation [47].
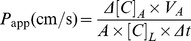



The P_app_ values were presented as percent of non treated control groups.

### Immunohistochemistry

Cell-cell connections and morphology of hCMEC/D3 cells were confirmed by immunostaining for junctional proteins β-catenin and claudin-5. Cells cultured on rat tail collagen coated Transwell inserts were washed in phosphate buffered saline (PBS) and fixed with acetone-methanol (1∶1) for β-catenin and with ethanol-acetic acid (95∶5) for claudin-5 at −20°C for 10 minutes. After rehydrating with PBS containing 1% fetal bovine serum and washing with PBS cells were blocked with 3% BSA in PBS at room temperature for 30 minutes. Samples were incubated overnight at 4°C with anti-β-catenin or anti-claudin-5 primary antibodies (Invitrogen, Life Technologies Corp., Carlsbad, USA; 1∶200). Incubation with Cy3-labeled or Alexa Fluoro 488-labeled anti-rabbit IgG secondary antibodies and bis-benzimide (Hoechst dye 33342) to stain cell nuclei lasted for 1 hour. Between and after incubations cells were washed three times with PBS. Inserts were mounted in Gel Mount (Biomeda, USA) and staining was examined by Olympus Fluoview FV1000 and Leica SP5 confocal laser scanning microscopes (Leica Microsystems GmbH, Wetzlar, Germany).

### Holographic Phase Contrast Microscopy

Digital holographic images were taken with a Holo-Monitor M3 instrument (Phase Holographic Imaging AB, Lund, Sweden). Endothelial cells were cultured on collagen coated culture dishes with borosilicate glass bottom (MatTek, Ashland, MA, USA). All treatments lasted for 4 hours. Holographic images of the same culture area were captured before and during treatments. Cell morphological changes were analysed by the Holostudio 2.4 software provided with the microscope (Phase Holographic Imaging AB, Lund, Sweden). Each point in the box plot reflects the data obtained on a single cell [48], [49].

### Statistical Analysis

All data presented are means ± SD or SEM as indicated in the text. The values were compared using the analysis of variance followed by Dunnett or Bonferroni posthoc tests using GraphPad Prism 5.0 software (GraphPad Software Inc., San Diego, CA, USA). Changes were considered statistically significant at *p*<0.05. All experiments were repeated at least three times, the number of parallel wells or inserts for each treatment and time point varied between 3 and 16.

## Results

### Methylglyoxal-induced Time- and Dose-dependent Cellular Toxicity Measured by Real-Time Impedance Monitoring

**Figure 1:**
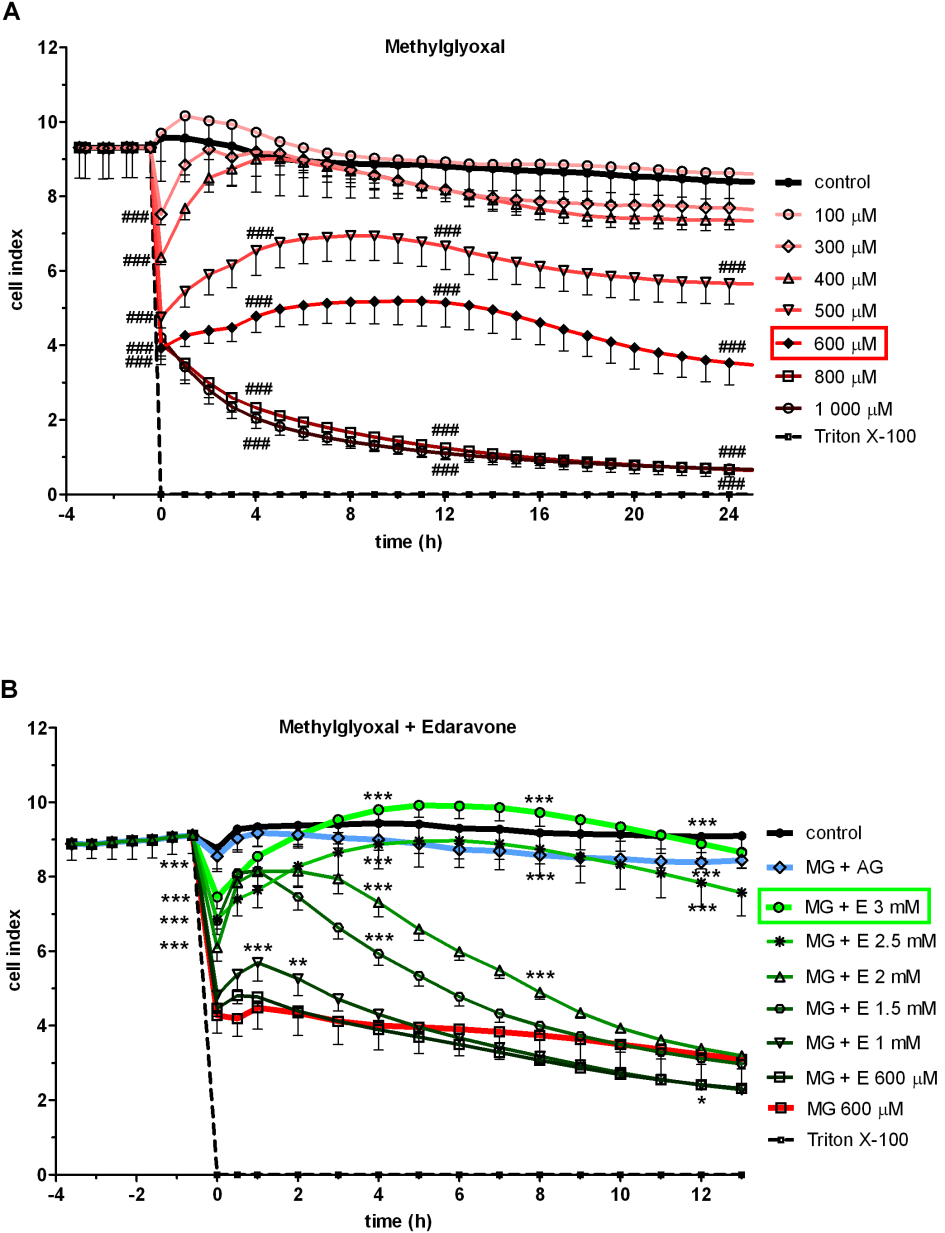
Effect of methylglyoxal and edaravone on cell viability. Effect of methylglyoxal (100–1000 µM) on human hCMEC/D3 endothelial cells measured by real-time cell electronic sensing method (A). Effect of co-treament with 600 µM methylglyoxal and different concentrations of edaravone (MG + E; 600–3000 µM) or aminoguanidine (MG + AG; 2 mM) (B). Cell index is expressed as an arbitrary unit and calculated from impedance measurements between cells and sensors. Data are presented as means ± SD, n = 10. Triton X-100 was used at 10 mg/mL concentration. Statistical analysis: two-way ANOVA followed by Bonferroni test. Statistically significant differences (*p*<0.05) from the control group (#) and from the methylglyoxal-treated group (*) are indicated.

The direct effect of methylglyoxal on cellular changes and cell viability was investigated by real-time impedance monitoring with an RTCA-SP instrument. Methylglyoxal showed dose and time-dependent toxicity in hCMEC/D3 endothelial cells (Figure 1A). The highest concentrations of methylglyoxal (300–1000 µM) caused a very quick and significant decrease of cell index values compared to control. However, this initial impedance drop was compensated with time depending on the amount of methylglyoxal. In case of cells treated with 100–400 µM methylglyoxal no effect on cell index was measured from 3 to 24 hour. A significant toxicity of methylglyoxal was seen in concentrations of 500 µM and above. Irreversible cell damage was observed at 800 and 1000 µM (Figure 1A).

To validate our data, 600 µM methylglyoxal concentration was tested using the colorimetric end-point MTT assay at 8-hour time point. The cell viability values after methylglyoxal treatment measured by the MTT test decreased to 56.7±4.6% (mean ± SD, n = 50) in good agreement with the decrease of cell index at the same concentration of methylglyoxal at the same time point (58.2±5.8%, mean ± SD, n = 10). The 600 µM concentration of methylglyoxal causing approximately 50% toxicity was selected for further tests.

To support the relevance of our data on endothelial cell line, the effect of methylglyoxal was also tested on cultures of primary rat brain endothelial cells (for preparation of cultures see Text S1). The toxicity of methylglyoxal was investigated by the WST-8 colorimetric assay of cell metabolism and LDH release reflecting cell membrane damage. No effect on viability was measured in primary cells treated with 100–300 µM methylglyoxal compared to control. Toxic effect was seen at 500 µM: the cell viability values decreased to 68.0±15.5% of the control (mean ± SEM, n = 8) by WST-8 assay and 76.7±5.9% (mean ± SEM, n = 12) by LDH assay (Figure S2).

### Co-administration of Edaravone Protects against Methylglyoxal-Induced Toxicity Measured by Real-Time Impedance Monitoring

To investigate the protective effects of edaravone on methylglyoxal-induced cell injury, brain endothelial cells were co-treated with different concentrations of edaravone and with a fixed concentration of methylglyoxal (600 µM). Cell index decreased by half in the methylglyoxal group (Figure 1B). Edaravone protected cells at 1000–3000 µM concentrations and reversed or attenuated the drop in cell index caused by methylglyoxal in a dose and time-dependent way. In case of cells co-treated with 60–600 µM edaravone no effect on cell index was measured compared to the methylglyoxal group. Edaravone at 1 mM showed a short term (0.5–2 hour) but statistically significant effect. The 1.5 and 2 mM concentrations of edaravone could protect the endothelial cells till 6 and 8 hours post-treatment, respectively. Long lasting protection of hCMEC/D3 cells could be observed at 2.5 and 3 mM concentrations of edaravone.

Our reference compound, aminoguanidine at 2 mM concentration showed a complete protection against methylglyoxal-induced cellular toxicity (Figure 1B). This result is in agreement with its effect determined by MTT test. Aminoguanidine at 2 mM concentration was found to be the most effective against methylglyoxal-induced cell damage in the colorimetric assay: cell viability was 93.2±0.6% of control at 8 hour time point. To determine the effect of protective agents alone, cells were incubated with different concentrations of edaravone (600–3000 µM) and aminoguanidine (600–2000 µM). There was no statistically significant decrease in cell viability measured by MTT assay and impedance monitoring in cells treated with edaravone or aminoguanidine alone. (Figure S1). Edaravone at 3 mM concentration increased both the impedance of the endothelial layers and the metabolic activity measured by MTT assay (121.8±5.9%, mean ± SD, n = 4, *p*<0.05).

Based on these results, the 3 mM concentration of edaravone showing the best protection of brain endothelial cells was selected for other investigation.

### Methylglyoxal-induced Time-Dependent ROS Production

**Figure 2:**
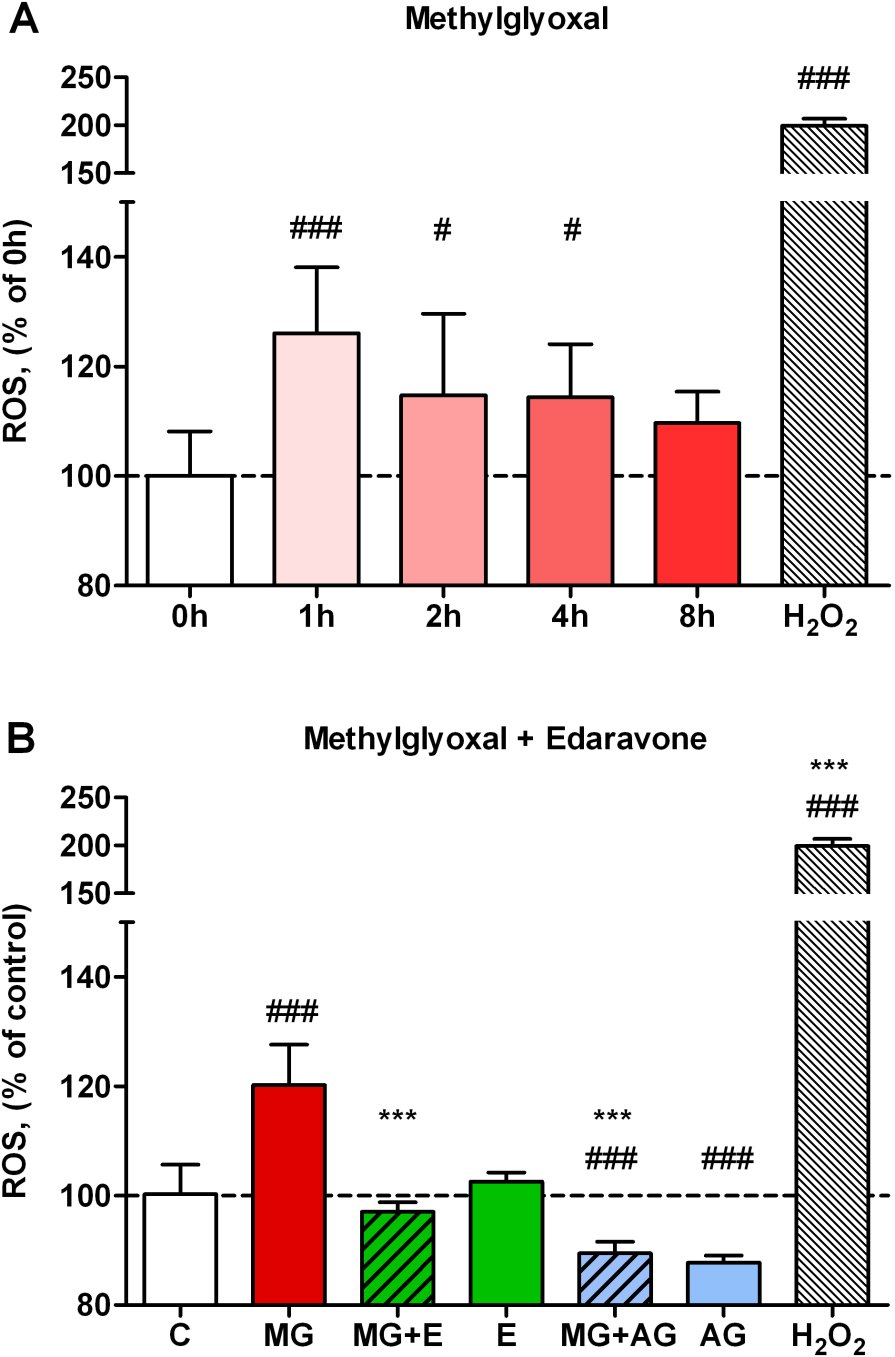
Effect of methylglyoxal and edaravone on reactive oxygen species production. Time-dependent effect of methylglyoxal (MG; 600 µM) on ROS production of human brain endothelial cells (hCMEC/D3) (A). Effect of co-treament with 600 µM methylglyoxal and 3 mM edaravone (MG + E) or 2 mM aminoguanidine (MG + AG) after 4 hours (B). Fluorescent intensity of ROS expressed as a percentage of control (C). Values presented are means ± SD, n = 9. Statistical analysis: ANOVA followed by Dunnett test. Statistically significant differences (*p*<0.05) from the control group (#) and from the methylglyoxal-treated group (*) are indicated.

As methylglyoxal generates not just carbonyl [18] but also oxidative stress [23], [24], ROS production in cells treated with 600 µM methylglyoxal was investigated at different time points (Figure 2A). The fluorescence intensity in cultured human brain endothelial cells measured by DCFDA assay at 1 hour time point was considered as 100% (1285.6±105.2, fluorescence intensity in arbitrary units). ROS production was significantly enhanced (126.1±12.0%, *p*<0.001) after one hour treatment by methylglyoxal as compared to control group, and remained elevated (114.7±14.9% and 114.4±9.7%) at 2 and 4 hours time points, respectively. ROS production in endothelial cells treated for 8 hours by methylglyoxal (109.7±5.7%) did not differ significantly from non-treated group. Hydrogen peroxide treatment (100 µM, 15 min), a positive control in the ROS assay, elevated the amount of ROS measured in brain endothelial cells by 2 fold (199.4±7.1%; Figure 2A). Based on these data and results from real-time monitoring, the 4 hours time point of 600 µM methylglyoxal treatment causing significantly elevated ROS production was chosen for further experiments.

### Co-administration of Edaravone Protects against Methylglyoxal-induced ROS Production

Edaravone (3 mM) completely inhibited the methylglyoxal-induced increase in ROS production (120.9±7.3%,) bringing back to the level of control (97.2±1.7%, *p*<0.001 as compared to the methylglyoxal treated group) at the 4 hours time point. Co-administration of aminoguanidine (2 mM) also decreased ROS production stimulated by methylglyoxal (89.1±2.1%). In contrast to edaravone, which had no effect alone (102.5±1.7%), treatment with aminoguanidine alone decreased the amount of ROS in endothelial cells below the level of the control group (87.4±1.3%; Figure 2B).

### Methylglyoxal-Induced Decrease of Resistance

**Figure 3:**
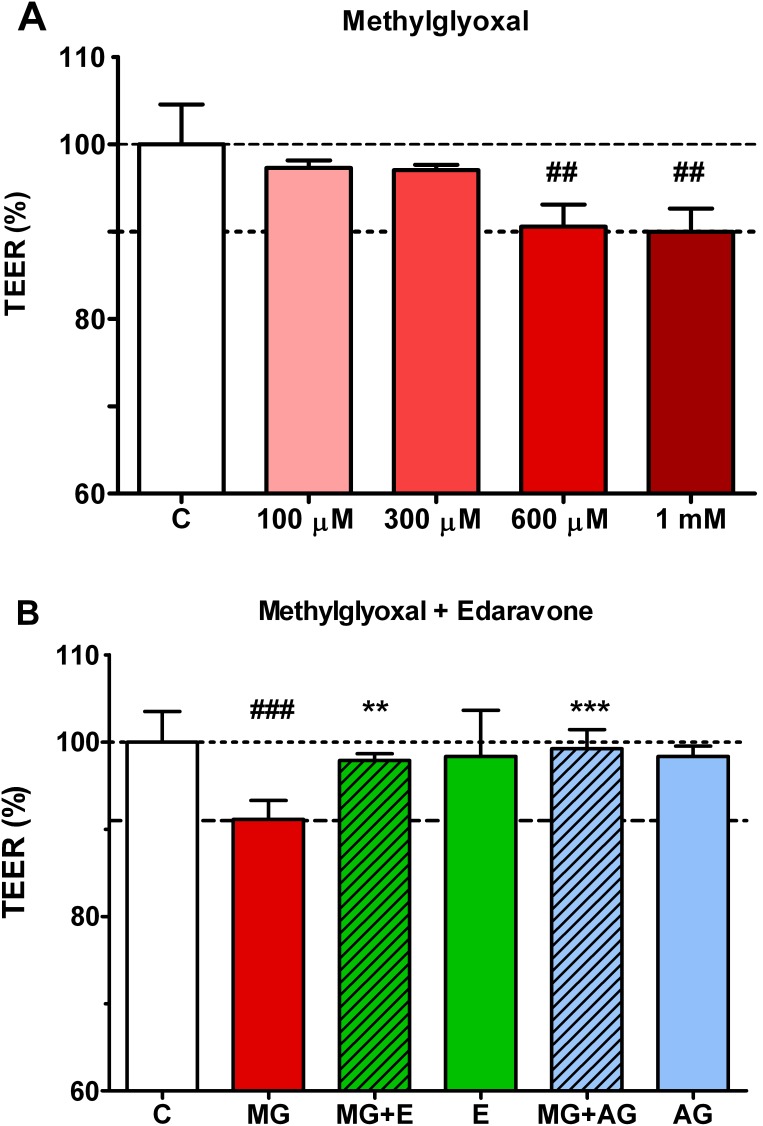
Effect of methylglyoxal and edaravone on the resistance of human brain endothelial monolayers. The effect of methylglyoxal on the resistance of human brain endothelial cells (hCMEC/D3) after 4-hours treatment (A). Protective effect of 3 mM edaravone (MG + E;) and 2 mM aminoguanidine (MG + AG) on methylglyoxal (MG, 600 µM) induced changes in the resistance of cell layers (4 hours co-treatment), and effect of 3 mM edaravone (E) and 2 mM aminoguanidine (AG) alone (B). TEER values are expressed as percentage of control (C). Values presented are means ± SD, n = 4. Statistical analysis: ANOVA followed by Dunnett test. Statistically significant differences (*p*<0.05) from the control group (#) and from the methylglyoxal treated group (*) are indicated.

The direct effect of different concentrations of methylglyoxal on the ionic permeability of brain endothelial monolayers was determined by TEER measurement. The resistance of hCMEC/D3 control group was 37.5±5.5 Ω×cm^2^ (mean ± SD; n = 14). Methylglyoxal at 100 and 300 µM concentrations (4 hours) did not cause significant changes. Treatment of endothelial cell monolayers with methylglyoxal at 600 µM and 1 mM concentrations significantly impaired the barrier integrity and decreased TEER to 90.6±2.5% and 89.9±2.7% (*p*<0.01 for both treatment groups) compared to control (Figure 3A). Treatment of primary brain endothelial cells with methylglyoxal at 300 and 500 µM concentrations also damaged the integrity of the barrier (Figure S3A). Methylglyoxal at 300 and 500 µM concentrations decreased the resistance (80.2±4.9% and 56.8±4.6%, respectively) as compared to the control (96.1±11.4%; n = 14).

### Co-administration of Edaravone Protects against Methylglyoxal-Induced Resistance Decrease

Co-administration of the protective agents with methylglyoxal preserved the barrier tightness and elevated resistance values to the level of control (edaravone: 97.9±0.8%; aminoguanidine: 99.2±2.1%). There was no significant difference between the resistance value of control and the groups treated with edaravone (3 mM) or aminoguanidine (2 mM) alone (Figure 3B).

### Methylglyoxal-Induced Dose-Dependent Permeability Increase

**Figure 4:**
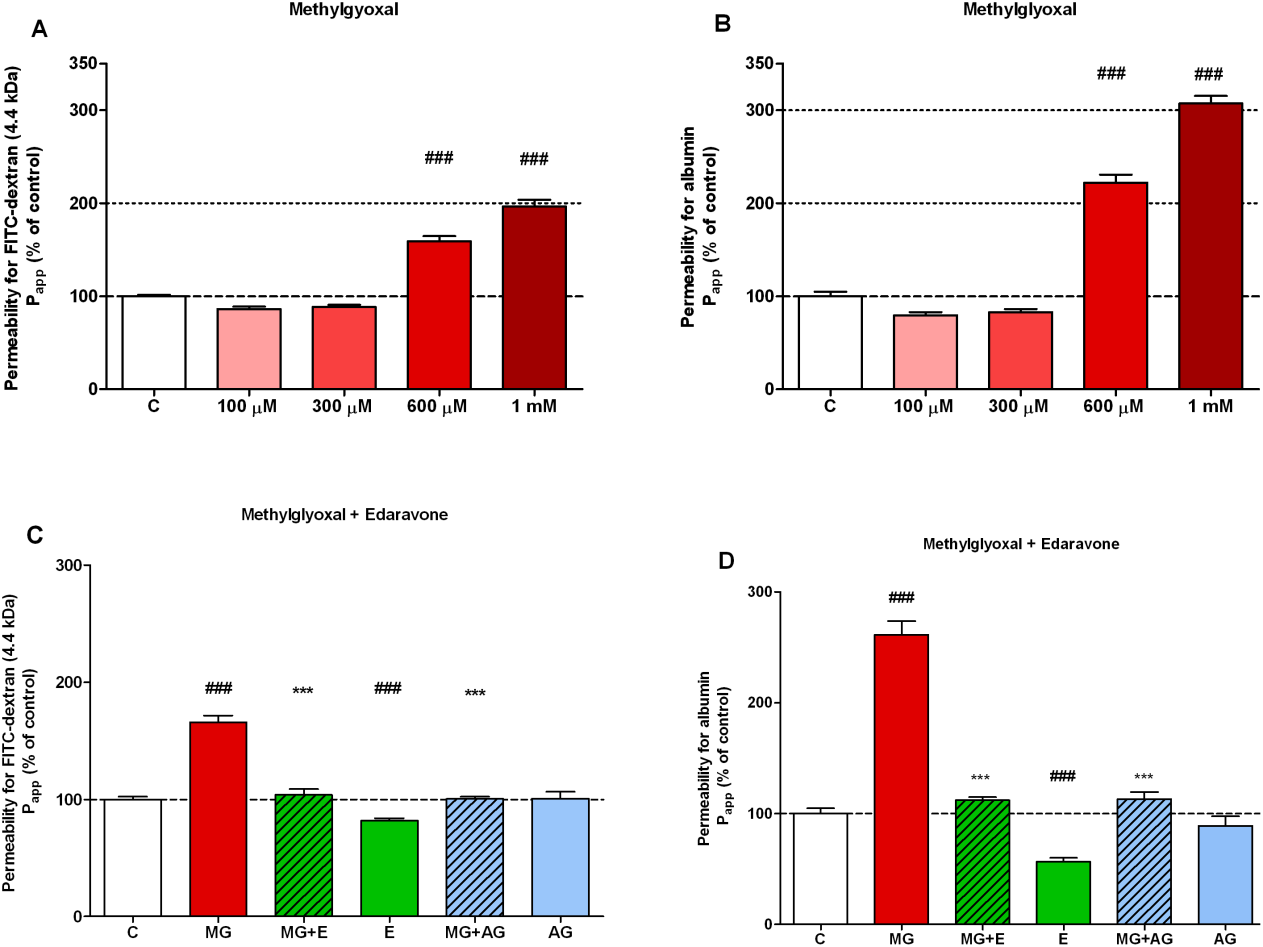
Effect of methylglyoxal and edaravone on the permeability of human brain endothelial monolayers. Dose-dependent effect of methylglyoxal-induced changes in the permeability of human brain endothelial cells (hCMEC/D3) for FITC-dextran (4.4 kDa) (A) and Evans blue labelled albumin (65 kDa) after 4 hours treatment (B). Protective effect of 3 mM edaravone (MG + E;) and 2 mM aminoguanidine (MG + AG) on methylglyoxal-induced (MG, 600 µM) changes in the permeability for FITC-dextran (4.4 kDa) (C) and Evans blue labelled albumin (D), when co-treated for 4 hours. Effect of 3 mM edaravone (E) and 2 mM aminoguanidine (AG) alone (C, D). Papp, apparent permeability coefficient expressed as a percentage of control (C). Values presented are means ± SD, n = 4. Statistical analysis: ANOVA followed by Dunnett test. Statistically significant differences (*p*<0.05) from the control group (#) and from the methylglyoxal treated group (*) are indicated.

Following the real-time impedance monitoring experiments, the direct effect of different methylglyoxal concentrations on the permeability of brain endothelial monolayers was measured using dextran (Figure 4A) and albumin (Figure 4B) as marker molecules. Cells were incubated with methylglyoxal (100–1000 µM, 4 hours), then permeability measurements were performed. Methylglyoxal at 100 and 300 µM concentrations did not cause barrier disruption. Treatment of hCMEC/D3 endothelial cell monolayers with methylglyoxal at 600 µM and 1 mM concentrations significantly impaired the barrier integrity and increased the permeability for both markers. The flux of the paracellular marker 4.4 kDa FITC-dextran was elevated by 1.5 fold (158.9±5.8%) in monolayers treated with 600 µM methylglyoxal and was doubled (196.4±7.2%) by 1 mM methylglyoxal treatment as compared to the control group (Figure 3A). The permeability coefficient values for transcellular permeability marker albumin (67 kDa) were one sixth of the values for FITC-dextran (4.4. kDa), 0.6±0.03×10^6 ^cm/s and 3.6±0.8×10^6 ^cm/s, respectively, in agreement with literature data [39]. The permeability for albumin was doubled (221.8±8.7%) after incubation with 600 µM methylglyoxal and tripled (307.2±8.1%) in the case of 1 mM methylglyoxal treatment (Figure 4B). Treatment of primary brain endothelial cells with 500 µM methylglyoxal also increased the permeability of monolayers for fluorescein (209.69±17.7%) and albumin (168.9±38.6%), but 100–300 µM concentrations had no effect (Figure S3B and S3C).

These results, that low concentrations of methylglyoxal (100–300 µM) do not increase permeability, while higher concentrations significantly reduce barrier integrity are in agreement with our results from impedance measurements. In further permeability assays to test the effect of protective agents, human brain endothelial cells were treated with 600 µM concentration of methylglyoxal.

### Co-administration of Edaravone Protects against Methylglyoxal-Induced Permeability Increase

Co-administration of edaravone at 3 mM concentration, selected previously by impedance measurement, could completely protect brain endothelial monolayers from the damaging effects of methylglyoxal (Figure 4C and 4D). Co-administration of edaravone shielded the cells from the damaging effect of methylglyoxal and kept the permeability for FITC-dextran at the control level (MG+E: 103.8±4.9% vs. MG: 165.6±6.0%). The same effect was seen by the co-administration of aminoguanidine at 2 mM concentration, endothelial permeability stayed at the level of control group (100.6±1.7%) (Figure 4C). The increased permeability for albumin of brain endothelial cell layers treated with methylglyoxal (261.3±12.2%) was completely prevented by the co-administration of 3 mM edaravone (112.1±2.8%) or 2 mM aminoguanidine (113.0±6.5%) (Figure 4D).

Aminoguanidine (2 mM) alone had no effect on the permeability of brain endothelial monolayers. Edaravone (3 mM) was able to tighten the barrier for both FITC-dextran (81.9±2.0%; Figure 4C) and albumin (56.5±3.5%; Figure 4D) as compared to the permeability of the control group.

### Co-administration of Edaravone Protects against Methylglyoxal-Induced Changes in Immunostaining

**Figure 5:**
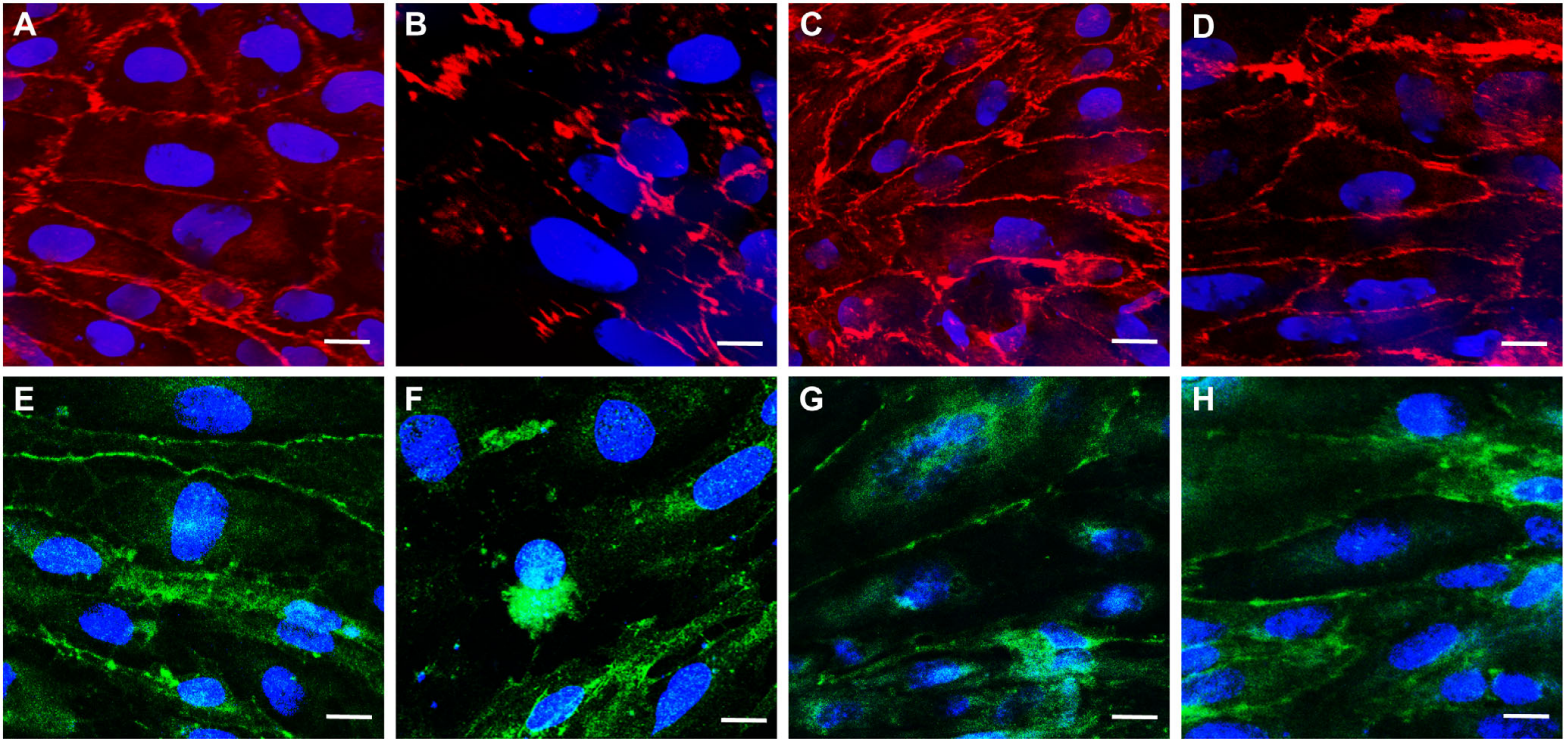
Effect of methylglyoxal and edaravone on junctional morphology. Immunostaining for adherens junction protein β-catenin (A–D) and integral tight junction membrane protein claudin-5 (E–H) in human hCMEC/D3 brain endothelial cells. Control (A and E), 4 hour treatment with methylglyoxal (600 µM; B and F), co-treatment with edaravone (3 mM; C and G) or aminoguanidine (2 mM; D and H). Blue color: bis-benzimide staining of cell nuclei; red color: immunostaining for β-catenin; green color: immunostaining for claudin-5. Bar = 20 µm.

The effect of methylglyoxal on cell-cell adhesion was investigated by immunostaining for β-catenin, a cytoplasmic adherens junction protein, and claudin-5, a transmembrane tight junction protein (Figure 5). In control monolayers β-catenin staining was localized to the cell border and the tightly apposed, elongated endothelial cells were well delineated (Figure 5A). Claudin-5 localization at the interendothelial junctions was less stringent than for β-catenin but clearly visible in part of the cell borders (Figure 5E) and resembled the immunostaining shown in the paper describing this cell line [39]. The pattern of the staining was dramatically changed in methylglyoxal (600 µM, 4 hours) treated cells. Treatment with methylglyoxal resulted in decreased immunostaining intensity, fragmentation or loss of the continuous cortical staining pattern, the appearance of intercellular gaps (Figure 5B and 5F) and apoptotic cells (Figure 5F). Co-administration of protective agents, edaravone (Figure 5C and 5G) and aminoguanidine (Figure 5D and 5H) attenuated these changes, the monolayer integrity was better preserved and the immunostaining pattern resembled to the control ones.

### Co-administration of Edaravone Protects against Methylglyoxal-Induced Morphological Changes Examined by Holographic Phase Contrast Microscopy

**Figure 6:**
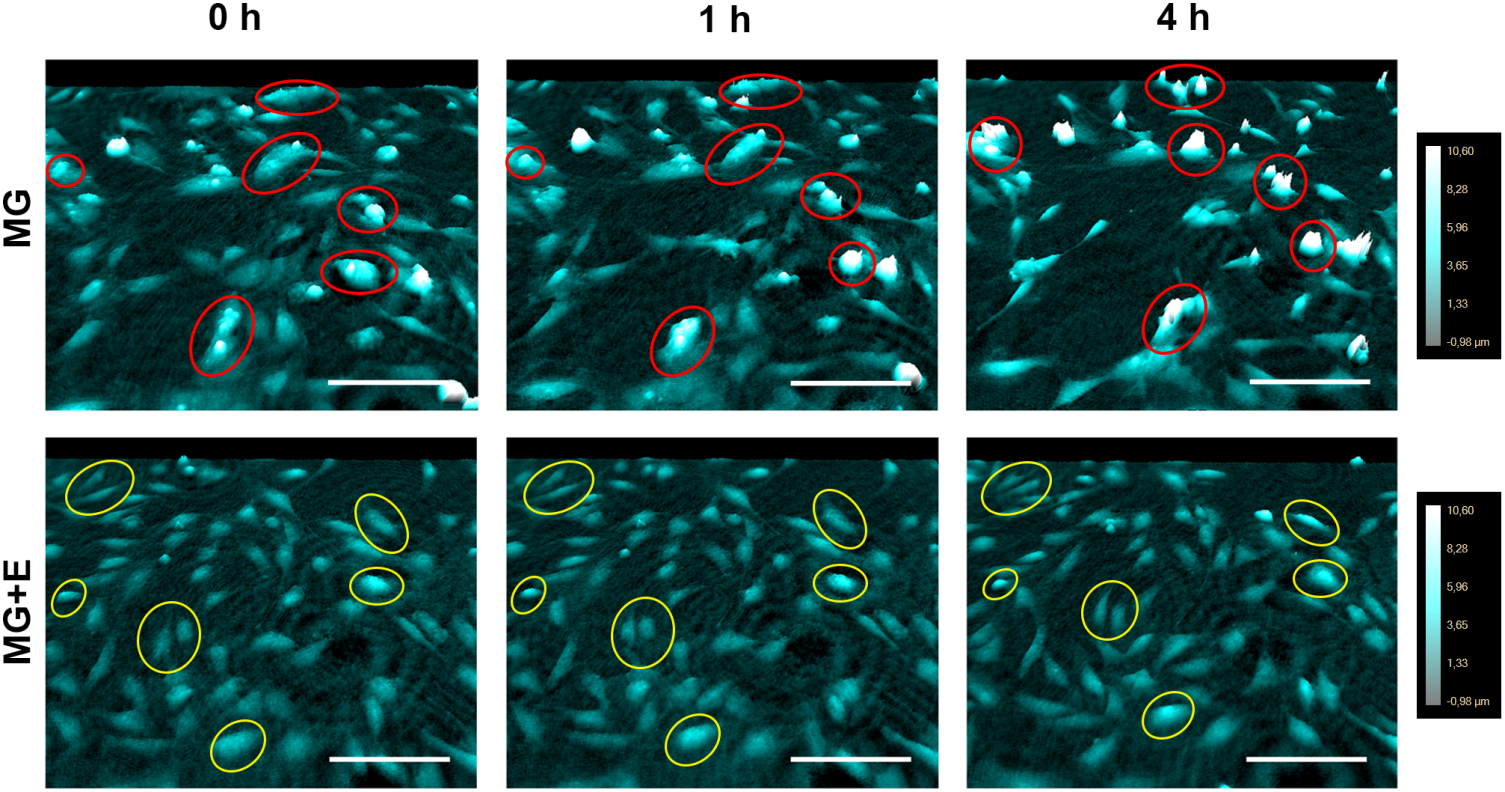
Effect of methylglyoxal and edaravone on cellular morphology. Holographic phase contrast images of morphological alterations induced in hCMEC/D3 human brain endothelial cells by treatment with methylglyoxal (MG; 600 µM) and co-treatment with 3 mM edaravone (MG + E) for 4 hours. Color scale bar correspond to the height of single cells. Data were analysed by means of HoloStudio 2.4 software. Red circles indicate cells with drastical changes in cell morphology. Yellow circles indicate cells without any morphological changes. Bar = 100 µm.

**Figure 7:**
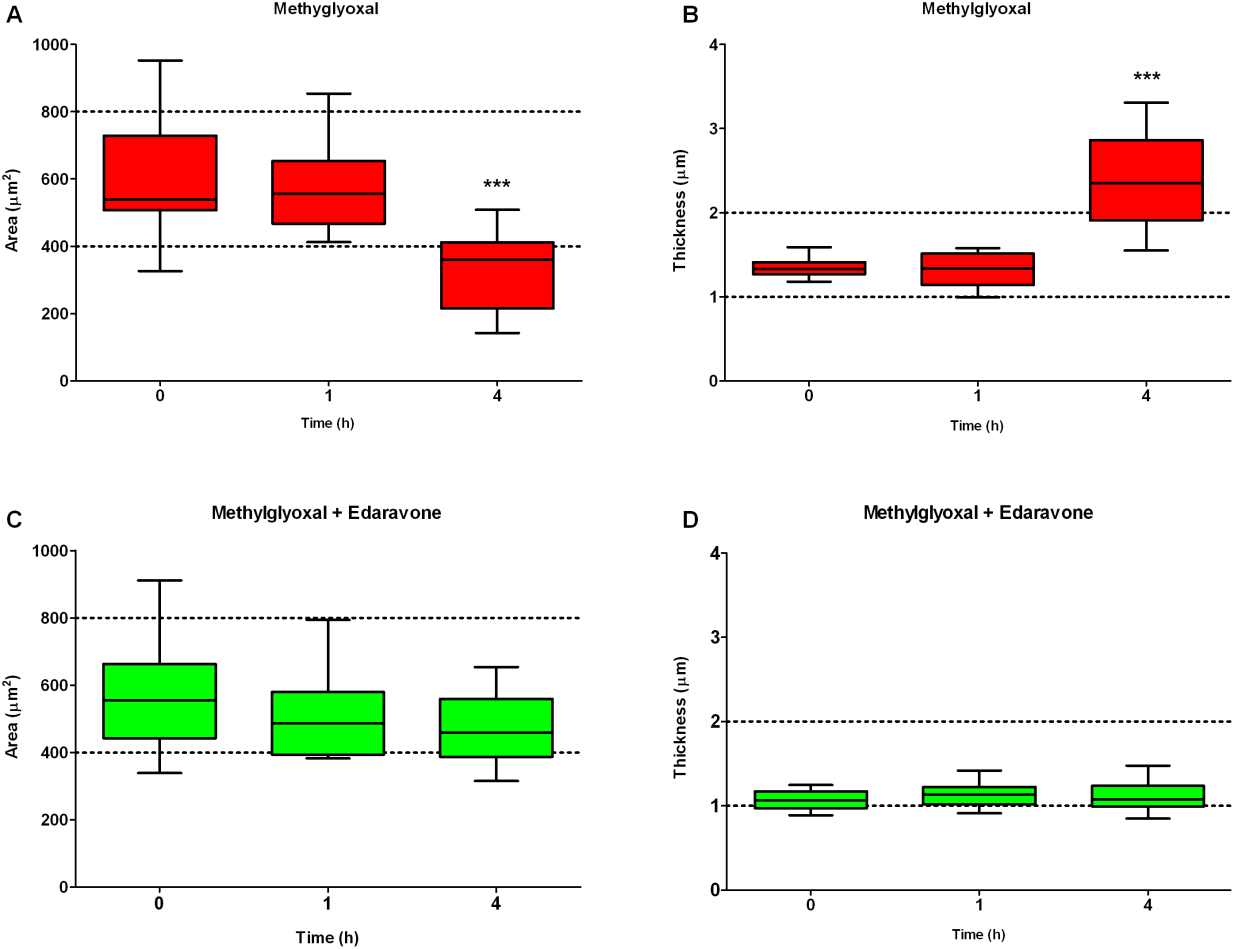
Effect of methylglyoxal and edaravone on area and thickness of adjacent cells. Morphological alterations induced in human brain endothelial cells (hCMEC/D3) by treatment with methylglyoxal at 600 µM (A and B) and co-treatment with edaravone at 3 mM (C and D) for 4 hours. Surface area (A and C) and average optical thickness (B and D) were calculated before and during treatments. Box represents 25 and 75 percentiles. Horizontal line represent the median. Whiskers show minimum and maximum values. Statistical analysis: ANOVA followed by Dunnett test, n = 12. Statistically significant differences (*p*<0.05) from 0 time point (*) are indicated.

Holographic phase contrast microscopic analysis was performed to visualize the morphological changes caused by methylglyoxal and the protective effect of edaravone (Figure 6). This novel technology enabled us to follow living cells in a label-free and non-invasive way. Morphological parameters of treated cells, such as surface area, optical thickness and cell volume could be measured. Holographic images were taken every 30 minutes before and during the 4-hour treatment of hCMEC/D3 cells. Endothelial cells show a flat, elongated shape and grow next to each other. Treatment with methylglyoxal caused drastic changes in cell morphology: as indicated by the colour-scale, growth of cell height was especially prominent (Figure 6). In contrast, in endothelial cells co-treated with edaravone (3 mM) and methylglyoxal (600 µM) there was no change in cell morphology during the treatment period (Figure 6). Two short videos on the cell morphology in both treatment groups at all timepoints are shown as supplementary data (Video S1 and S2). The analysis of morphological data is shown on Figure 7. During treatment with methylglyoxal the area of the cells significantly decreased (63.1±33.7%) and their optical thickness increased by 1.8 fold (176.2±36.1%) compared to the values at the beginning of treatment. These data indicate that endothelial cells treated with methylglyoxal contracted and rounded up, which is also visible on Figures 6 and Video S1. The volume of the cells was unchanged. Meanwhile, no changes were observed in cells co-treated with edaravone and methylglyoxal (Figure 7) or in the control group which was treated with medium only. This is the first report on methylglyoxal-induced morphology changes in brain endothelial cells using holographic phase contrast imaging.

## Discussion

### Carbonyl stress and brain endothelial damage

Higher incidence of stroke, dementia and Alzheimer’s disease is observed in diabetes mellitus [50], [51]. Carbonyl stress induced by high level of methylglyoxal is responsible for the diabetes related vascular complications [52]. A direct toxic effect of methylglyoxal on brain microvessels was proposed in a recent study [28]. In the present study we further supported the fact that methylglyoxal alone could induce damage to brain endothelial cells. Methylglyoxal exerted a time- and dose-dependent toxicity on cultured human brain endothelial cells; it significantly reduced the integrity of the barrier measured by both functional and morphological experiments. This is the first study to provide kinetic data on the toxicity of methylglyoxal by impedance-based cell electronic sensing, a noninvasive label-free technique. The two different cell viability assays we used were in complete agreement on the direct cellular damaging effect of methylglyoxal, impedance data reflecting changes in cell adhesion, cell shape and number were confirmed by MTT tests measuring the metabolic activity of cells. Our data lend support to and expand previous findings on the effect of methylglyoxal on human brain endothelial cells [25], [28], [37], [53]. We selected the human hCMEC/D3 cell line as a simplified model of the blood-brain barrier. This cell line is widely used in different experiments, including pharmacological and drug studies [40]. To support the relevance of our data on hCMEC/D3 endothelial cell line, the effect of methylglyoxal was also tested on primary cultures of rat brain endothelial cells. The observed effects were in agreement with our observations on the human cell line, indicating a similar sensitivity of primary endothelial cells and hCMEC/D3 endothelial cell line for the toxic effects of methylglyoxal. We found no data on primary brain endothelial cells related to methylglyoxal in the literature, therefore the present observation is the first study to include primary brain endothelial cells in this setting. The relevance of our findings on endothelial cells is limited by the use of high concentrations of methylglyoxal to induce barrier damage, a common concern in cell culture studies. However, in four recent and independent studies both the time needed to measure methylglyoxal-induced injury in cultured endothelial cells and the concentrations used were in similar range as in our study [25], [28], [37], [53].

Damage by methylglyoxal is mediated not only via carbonyl stress, but also by oxidative stress. [23], [24]. Reactive oxygen species are generated as by-products of protein glycation [54]. Furthermore, methylglyoxal increases glycation of selected mitochondrial proteins resulting in increased formation of superoxide [55]. Elevated level of ROS weakens the barrier integrity [56], however the contribution of methylglyoxal-triggered ROS production in the increased endothelial permeability is controversial [28]. In the present study we confirmed that methylglyoxal treatment promotes oxidative stress in brain endothelial cells, similarly to previous studies on endothelial cells [28], [53] and other cellular systems [57]. The kinetics of ROS production also helped to determine the optimal time point for protection assays and other experiments: a time point, where ROS formation was still elevated, was purposefully selected.

In good agreement with the data from toxicity measurements methylglyoxal increased the permeability of human and rat brain endothelial monolayers. The effect was dose-dependent, with only high concentrations of methylglyoxal causing significant damage in barrier integrity. These data are in accordance with findings of a previous study showing that methylglyoxal induced permeability increase in brain endothelial cells [28]. In that study decreased transendothelial electrical resistance and increased dextran flux were coupled with glycation of occludin and disturbed localization of zonula occludens-1 tight junction proteins [28]. In the present study we found the redistribution of two other junctional proteins important for the regulation of brain endothelial permeability, namely the tight junction protein claudin-5 and adherens junctional protein β-catenin in methylglyoxal-treated brain endothelial cells. Claudin-5 is the most abundant integral membrane protein of the brain endothelial tight junctions and a major regulator of the blood-brain barrier permeability [59]. β-catenin binds the cytoplasmic tail of vascular endothelial cadherin (VE-cadherin) to the actin cytoskeleton [58]. Besides its role as a cytoskeletal linker, β-catenin is an important signaling and transcriptional factor at the blood-brain barrier regulating its tightness [59]. Delocalization/redistribution of β-catenin in brain endothelial cells is linked to permeability increase in many pathologies [60], [61], [62]. We demonstrated for the first time, that treatment with methylglyoxal resulted in fragmentation and loss of the continuous cortical pattern of β-catenin in brain endothelial cells, confirming the importance of β-catenin in barrier integrity.

For the maintenance of the integrity of the blood-brain permeability barrier the attachment of endothelial cells to the basement membrane is crucial [63]. Damage to the basal lamina weakens microvascular wall structures and results in increase of brain vessel permeability in vivo [64]. Our data confirmed these observations and visualized for the first time the kinetics of morphological changes caused by methylglyoxal using a novel technique, holographic phase contrast microscopy. This technology follows the morphology of living cells in a label-free and non-invasive way and detects only the effects of the tested compound [48], [49]. In our study methylglyoxal changed drastically the shape of brain endothelial cells by time: the area of cells decreased, their optical height significantly increased indicating cell-cell and cell-basal lamina detachment. Similar observation was made in case of peripheral endothelial cells, where modification of basement membrane by methylglyoxal caused cell detachment, anoikis and inhibition of angiogensis [65].

### Protective effect of edaravone on methylglyoxal-injured brain endothelial cells

Edaravone was reported to protect brain microvessels and the blood-brain barrier after ischemic stroke [33], [34]. In a recent study, edaravone reduced cell injury in oxygen-glucose deprivation in vitro model. Edaravone pretreatment attenuated methylglyoxal-induced decrease in cell viability of brain endothelial cells [37]. In our study co-treatment with edaravone provided a complete protection against the toxic effect of methylglyoxal. We could see a dose- and time-dependent effect based on kinetic data from impedance measurements. Our data are in agreement with viability studies on the effect of edaravone pretreatment against methylglyoxal-induced toxicity [37]. Edaravone treatment alone increased the metabolic activity and impedance of the endothelial layers. A beneficial effect of edaravone on human endothelial cells was also found previously by resistance measurement and proteomic assay [35], [36]. The edaravone concentrations used were in the millimolar range similarly to another independent work on cultured human brain endothelial cells. For long-lasting protection suprapharmacological concentration of edaravone [66] was needed in our culture study. However, the applied methylglyoxal levels were also comparably high, as in other in vitro methylglyoxal studies [25], [28], [37], [53]. Although we use higher concentrations in cultured cells, importantly, the ratio of the methylglyoxal to edaravone used in our study is the same as the ratio of the pathological plasma methylglyoxal concentrations [67] to clinical concentrations of edaravone [66].

Originally, edaravone has been described as a drug to treat ischemic stroke by protecting against oxidative stress [31]. Its antioxidant effect was observed in our experiment, too. In a recent independent study edaravone suppressed methyglyoxal-induced ROS production in human brain endothelial cells by two possible mechanisms [37]. Pre-treatment with edaravone decreased methylglyoxal-induced AGE accumulation and activation of its receptor RAGE [37], and the subsequent production of ROS [68], [69]. Furthermore, edaravone inhibited protein-glycation by methylglyoxal in a cell free system [37], therefore, it decreased ROS generated as by-products during protein glycation [54]. All these results together indicate that the antioxidant mechanisms induced by edaravone contribute to its protective effect against methylglyoxal-induced oxidative stress.

Prior work described, that edaravone protects from methylglyoxal-induced injury by inhibiting AGEs/RAGE/oxidative stress in human brain microvascular endothelial cells [37]. However, it remained unanswered whether edaravone can also protect against methylglyoxal-induced barrier dysfunction in brain endothelial monolayers. Therefore, this study focused on the protective effect of edaravone against methylglyoxal-induced barrier damage. We found that co-treatment with edaravone restored barrier properties of endothelial cells and protected against methylglyoxal-induced decrease of resistance and increase in permeability for paracellular and transcellular markers. Moreover, we also demonstrated that edaravone treatment alone tightened the brain endothelial barrier. Our data expand and further support previous observations on barrier enhancing effect of edaravone [35], [36].

Increased endothelial permeability was coupled with disturbed localization of junctional proteins claudin-5 and β-catenin after incubation with methylglyoxal, while co-treatment with edaravone restored distribution of both proteins along the cell borders. Similar observation was made in a previous study, where edaravone treatment enhanced β-catenin at cell-cell contact area and the cortical arrangement for its linked protein, actin on half confluent endothelial monolayer [35]. Our holographic phase contrast microscopic data are in accordance with these observations: edaravone completely prevented methylglyoxal-induced changes in cell morphology, no sign of detachment and cellular morphological change was observed, indicating there was no cytoskeletal rearrangement.

Our results have answered the question that edaravone can protect against methylglyoxal-induced barrier dysfunction in brain endothelial cells. However, the underlying signal transduction pathways during co-treatment were not examined and further work is needed to elucidate the precise mechanism.

## Conclusion

This is the first study to investigate the protection of cerebral endothelial cells by edaravone in co-treatment with methylglyoxal. Our results provide compelling evidence for barrier protective effect of edaravone in cultured endothelium of brain microvasculature. Data from this study could have therapeutical implication for disorders and diseases that are associated with carbonyl stress.

## Supporting Information

Figure S1 **Effect of edaravone and aminoguanidine on cell viability.** Effect of edaravone (E; 600 µM–3 mM) and aminoguanidine (AG; 600 µM–2 mM) on human hCMEC/D3 endothelial cells measured by real-time cell electronic sensing (RTCA-SP) method (A) and by MTT metabolic assay at 8 hours timepoint (B). MTT assay and cell index data are expressed as percentage of control. Data are presented as means ± SD, n = 6. Triton X-100 was used at 10 mg/mL concentration. Statistical analysis: ANOVA followed by Dunnett or by Bonferroni test. Statistically significant differences (*p*<0.001) from the control group (#) are indicated.
(TIF)

Figure S2 **Effect of methylglyoxal on the viability of primary brain endothelial cells.** Effect of methylglyoxal (100–1000 µM) on primary rat brain endothelial cells measured by WST-8 (A) and lactate dehydrogenase (LDH) release assay (B). Values are expressed as percentage of control. Data are presented as means ± SEM, n = 20. Statistical analysis: one-way ANOVA followed by Dunett test. Statistically significant differences (*p*<0.05) from the control (C) group (#) are indicated.
(TIF)

Figure S3 **Effect of methylglyoxal on the barrier properties of primary brain endothelial monolayers.** Dose-dependent effect of methylglyoxal-induced changes in the resistance (A) and the permeability of primary rat brain endothelial cells for sodium-fluorescein (B) and Evans blue labeled albumin (B). Transendothelial electrical resistance (TEER) and endothelial permeability coefficient (Pe) are expressed as a percentage of control (C). Data presented are means ± SEM, n = 16–24. Statistical analysis: ANOVA followed by Dunnett test. Statistically significant differences (*p*<0.05) from the control group (#) and from the methylglyoxal treated group (*) are indicated.
(TIF)

Text S1 **Materials and Methods for figures S2 and S3.**
(DOC)

Video S1 **Effect of methylglyoxal on cellular morphology.** Videos were made from holographic phase contrast images on morphological alterations induced in hCMEC/D3 human brain endothelial cells by treatment with 600 µM methylglyoxal (Video S1) and co-treatment with 3 mM edaravone (Video S2). Pictures were taken every 30 min until 4 hours. Color scale bar correspond to the height of single cells. Data were analysed by means of HoloStudio 2.4 software.
(AVI)

Video S2 **Effect of methylglyoxal on cellular morphology.** Videos were made from holographic phase contrast images on morphological alterations induced in hCMEC/D3 human brain endothelial cells by treatment with 600 µM methylglyoxal (Video S1) and co-treatment with 3 mM edaravone (Video S2). Pictures were taken every 30 min until 4 hours. Color scale bar correspond to the height of single cells. Data were analysed by means of HoloStudio 2.4 software.
(AVI)
