# Supplementary figures and images for: Edaravone Protects against Methylglyoxal-Induced Barrier Damage in Human Brain Endothelial Cells
Source: PLoS One. 2014 Jul 17;9(7):e100152. doi: 10.1371/journal.pone.0100152 (PMC4102474; doi:10.1371/journal.pone.0100152)

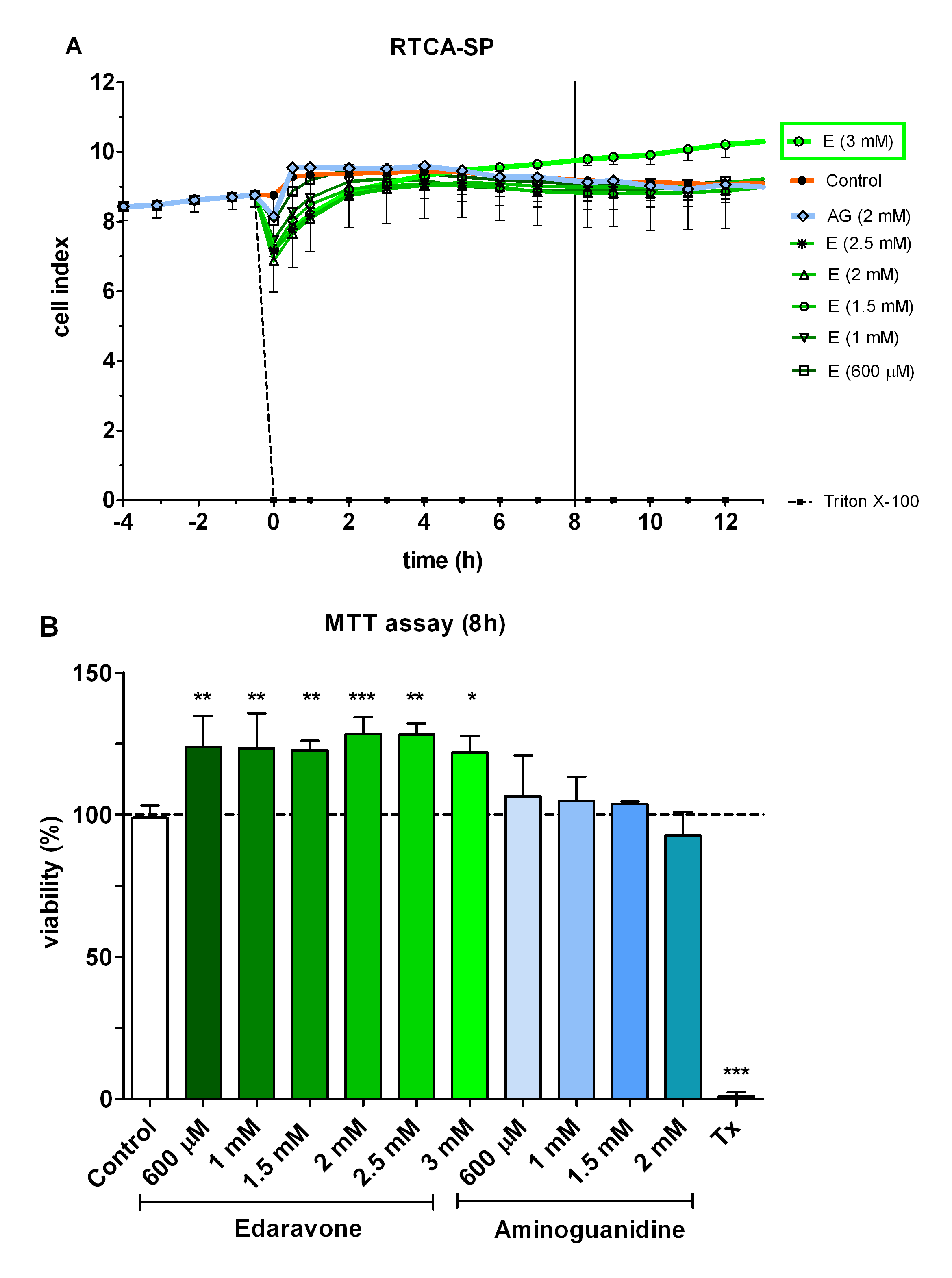

Supplement: Figure S1 — Effect of edaravone and aminoguanidine on cell viability. Effect of edaravone (E; 600 µM–3 mM) and aminoguanidine (AG; 600 µM–2 mM) on human hCMEC/D3 endothelial cells measured by real-time cell electronic sensing (RTCA-SP) method (A) and by MTT metabolic assay at 8 hours timepoint (B). MTT assay and cell index data are expressed as percentage of control. Data are presented as means ± SD, n = 6. Triton X-100 was used at 10 mg/mL concentration. Statistical analysis: ANOVA followed by Dunnett or by Bonferroni test. Statistically significant differences (p<0.001) from the control group (#) are indicated. (TIF) [file pone.0100152.s001.tif]

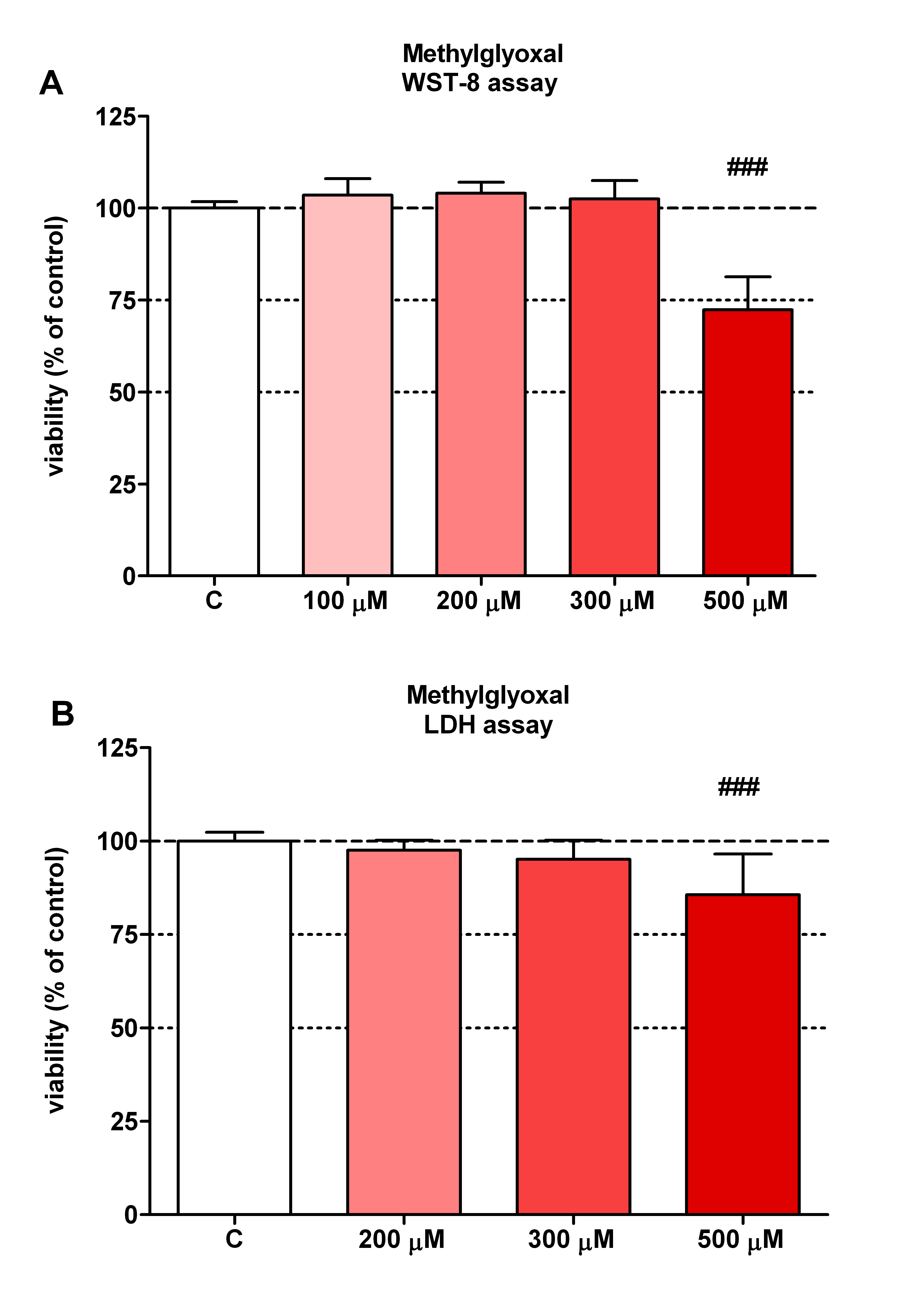

Supplement: Figure S2 — Effect of methylglyoxal on the viability of primary brain endothelial cells. Effect of methylglyoxal (100–1000 µM) on primary rat brain endothelial cells measured by WST-8 (A) and lactate dehydrogenase (LDH) release assay (B). Values are expressed as percentage of control. Data are presented as means ± SEM, n = 20. Statistical analysis: one-way ANOVA followed by Dunett test. Statistically significant differences (p<0.05) from the control (C) group (#) are indicated. (TIF) [file pone.0100152.s002.tif]

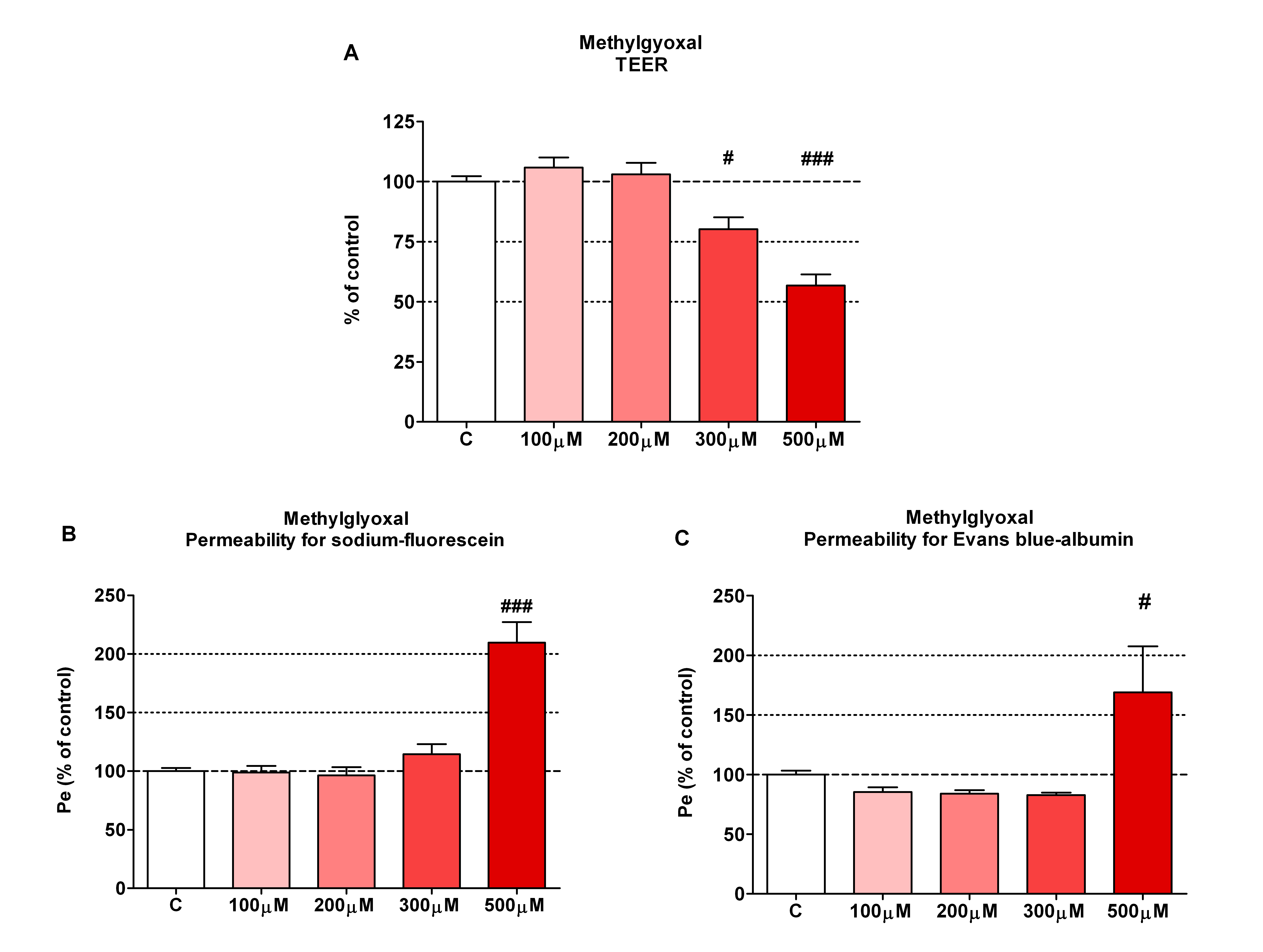

Supplement: Figure S3 — Effect of methylglyoxal on the barrier properties of primary brain endothelial monolayers. Dose-dependent effect of methylglyoxal-induced changes in the resistance (A) and the permeability of primary rat brain endothelial cells for sodium-fluorescein (B) and Evans blue labeled albumin (B). Transendothelial electrical resistance (TEER) and endothelial permeability coefficient (Pe) are expressed as a percentage of control (C). Data presented are means ± SEM, n = 16–24. Statistical analysis: ANOVA followed by Dunnett test. Statistically significant differences (p<0.05) from the control group (#) and from the methylglyoxal treated group (*) are indicated. (TIF) [file pone.0100152.s003.tif]
