# Supplementary material for: Edaravone Protects against Methylglyoxal-Induced Barrier Damage in Human Brain Endothelial Cells
Source: PLoS One. 2014 Jul 17;9(7):e100152. doi: 10.1371/journal.pone.0100152 (PMC4102474; doi:10.1371/journal.pone.0100152)
Supplement: Text S1 — Materials and Methods for figures S2 and S3. (DOC) [file pone.0100152.s004.doc]

# Text S1

# Materials and Methods for supplementary figures S2 and S3

All procedures involving experimental animals adhered to the law (No. 105) and notification (No. 6) of the Japanese Government, and were approved by the Laboratory Animal Care and Use Committee of Fukuoka University.

## Materials

All reagents were purchased from Sigma-Aldrich Ltd., unless otherwise indicated.

## Isolation of primary rat brain endothelial cells

Primary cultures of rat brain capillary endothelial cells (PRBECs) were prepared from 3 week-old Wistar rats CREA Japan, Inc. (Tokyo, Japan). The rats had free access to food and water and were maintained on a 12-h dark/light cycle in a room with controlled temperature (24-1ºC) and humidity (55-5%). After decapitation the meninges were carefully removed from the forebrains, and the gray matter was minced in DMEM cooled at 2ºC, and digested with collagenase type 2 (1 mg/mL, Worthington, Lakewood, NJ, USA) for 1.5 h at 37ºC. The pellet was separated by centrifugation in 20% bovine serum albumin (BSA)-DMEM (10006g, 20 min). The microvessels obtained in the pellet were further digested with collagenase/dispase (1 mg/mL, Roche, Mannheim, Germany) for 1 h at 37ºC. Microvessel clusters containing pericytes and endothelial cells were separated on a 33% continuous Percoll (GE Healthcare, Buckinghamshire, UK) gradient, collected and washed twice with DMEM before plating on collagen type IV-fibronectin (both 0.1 mg/mL) coated dishes. Rat brain endothelial cell cultures were maintained in DMEM/F12 medium supplemented with 10% plasma derived sera, basic fibroblast growth factor (1.5 ng/mL, Roche), heparin (100 µg/mL), insulin (5 µg/mL), transferrin (5 mg/mL), sodium selenite (5 ng/mL; insulin-transferrin-sodium selenite media supplement) and gentamicin (50 µg/mL)] containing puromycin (4 µg/mL) at 37ºC in a humidified atmosphere of 5% CO2/95% air, for two days. To remove the puromycin, cells were washed three times with fresh medium and cultured for three days. On the fifth day, cell culture typically reached 80–90% confluency.

## Treatments

Brain endothelial cells were treated with methylglyoxal in the 100-500 µM concentration range. Confluent primary rat brain endothelial cells were treated in DMEM/F12 medium supplemented with basic fibroblast growth factor (1.5 ng/mL, Roche), heparin (100 µg/mL), insulin (5 µg/mL), transferrin (5 mg/mL), sodium selenite (5 ng/mL; insulin-transferrin-sodium selenite media supplement) and gentamicin (50 µg/mL).

## Cell Viability Assays

For endpoint viability assays brain endothelial cells were grown to confluency in 96-well culture plates and treated with methylglyoxal, with or without protective agents for 8 hours. In WST-8 assay (Cell Counting Kit, Dojindo, Kumamoto, Japan) similarly to MTT assay, living cells produce formazan by mitochondrial dehydrogenase. The absorbance of the highly water-soluble formazan dye (WST-8) was measured in each sample at wavelengths of 450 nm (test wavelength) and 700 nm (reference wavelength). Cells receiving treatment medium without methylglyoxal or protective agents, the control group, was considered as 100 % viable.

Lactate dehydrogenase (LDH) release is the indicator of cell membrane damage. was determined from culture supernatants by a commercially available kit (Cytotoxicity detection kit LDH, Roche, Basel, Switzerland). After the treatment 50 μl samples from culture supernatants were removed and incubated with equal amounts of reaction mixture for 15 minutes. The enzyme reaction was stopped by 0.1 M HCl. Absorbance was measured at a wavelength of 492 nm with a microplate reader (Opsys MR, DYNEX Technologies, Chantilly, VA,USA). Cell death was calculated as percentage of the total LDH release from cells treated by 1 % (w/v) Triton X-100 detergent.

## Measurement of Barrier Functions: Transendothelial Electrical Resistance

Transendothelial electrical resistance (TEER) reflects the permeability of intercellular tight junctions for ions. TEER was measured an Epithelial-volt-ohm meter and Endohm-6 chamber electrodes (World Precision Instruments, Sarasota, FL, USA) and expressed relative to the surface area (Ω × cm^2^). TEER values of cell-free inserts were subtracted from the measured data. Resistance measurements were carried out before and after treatments to check the barrier integrity. The TEER values are presented as percent of non treated control groups.

## Measurement of Barrier Functions: Permeability Experiments

Cell culture inserts were transferred to 24-well plates containing 0.6 ml permeability assay buffer (141 mM NaCl, 2.8 mM CaCl_2_, 1 mM MgSO_4_, 4 mM KCl, 1 mM NaH_2_PO_4_, 10 mM glucose and 10 mM Hepes, pH 7.4) in the lower or abluminal compartments. In the inserts (luminal compartment), culture medium was replaced by 0.1 ml buffer containing 100 µg/ml Na–Fluorescein (MW: 376 Da) and 4% bovine serum albumin mixed with 0.67 mg/mL Evan’s blue dye (EBA; MW: 67,000 Da). Samples (400 µL) were removed from each side at 15, 30, 45, 60, 120 and 180 min. To ensure mixing of the layers, we stirred the assay buffer in the receiver chamber, into which test compounds permeate, with a pipette before removing the buffer and immediately replacing with fresh permeability assay buffer. The concentrations of Na–Fluorescein were determined with a CytoFluor Series 4000 fluorescence multiwall plate reader (PerSeptive Biosystems) using a fluorescein filter pair (Ex(λ) 485±10 nm; Em(λ) 530±10 nm). The EBA concentration in the abluminal chamber was measured by determining the absorbance of samples at 630 nm with a microplate reader (Opsys MR, DYNEX Technologies, Chantilly, VA,USA).

Transendothelial permeability coefficient (P_e_) was calculated from the transport of donor (luminal) compartment volume from which the tracer is completely cleared. Cleared volume was calculated from the concentration (C) of the tracer in the abluminal and luminal compartments and the volume (V) of the abluminal compartment (0.5 ml) by the following equation:

The average cleared volume was plotted *vs*. time, and permeability × surface area product value for endothelial monolayer (PS_e_) was calculated by the following formula:

PS_e_ divided by the surface area (1 cm^2^ for Transwell-12) generated the endothelial permability coefficient (P_e_; in 10^-3^ cm/min). The values were presented as percent of non treated control groups.

## Statistical Analysis

Data are presented as means ± SEM. The values were compared using the analysis of variance followed by Dunnett or Bonferroni posthoc tests using GraphPad Prism 5.0 software (GraphPad Software Inc., San Diego, CA, USA). Changes were considered statistically significant at *p* < 0.05. All experiments were repeated at least three times, the number of parallel wells or inserts for each treatment and time point varied between 3 and 6.
